# Geographical, Socioeconomic, and Gender Inequalities in Opioid Use in Catalonia

**DOI:** 10.3389/fphar.2021.750193

**Published:** 2021-10-21

**Authors:** Sara Serra-Pujadas, Cristina Alonso-Buxadé, Júlia Serra-Colomer, Júlia Folguera, Neus Carrilero, Anna García-Altés

**Affiliations:** ^1^ Facultat d’Economia i Empresa, Universitat Pompeu Fabra, Barcelona, Spain; ^2^ Agència de Qualitat i Avaluació Sanitàries de Catalunya (AQuAS), Barcelona, Spain; ^3^ CIBER de Epidemiología y Salud Pública (CIBERESP), Barcelona, Spain; ^4^ Institut d’Investigació Biomèdica (IIB Sant Pau), Barcelona, Spain

**Keywords:** opioids, gender, socioeconomic factors, prescriptions, medical bias, chronic pain

## Abstract

**Background:** In recent years, worldwide opioid use has seen a sharp increase, especially for the treatment of chronic non-cancer pain. Catalonia is no exception to this trend. However, no recent studies have addressed the socioeconomic and gender inequalities in opioid use in the different geographical areas of Catalonia.

**Methods:** We performed an ecological study to analyse the associations between socioeconomic status, gender and the use of opioids in the 372 Health Areas of Catalonia. Robust Poisson models were performed to analyse the data provided from the Central Register of Insured Persons and dispensing data from the Electronic Prescription Database.

**Results:** The results show that socioeconomic status has a major impact on opioid use, with the most deprived areas presenting the highest levels of use. There are major inequalities in the DDD/1,000 inhabitants per areas. Moreover, women have much higher utilization rates than men, especially in more deprived areas. The greatest difference is observed in the use of weak opioids in women: the DDD/1,000 inhabitants per day was 2.34 in the area with the lowest use, compared with 22.18 in the area with the highest use.

**Conclusions:** Our findings suggest that stronger action is needed to promote best practices in prescribing for chronic pain and to reduce socioeconomic and gender variation between geographical areas. This study provides a model for routine monitoring of opioid prescription for targeted interventions aimed at lowering high-dose consumption in specifically identified areas.

## Introduction

Pain is a growing public health priority. Demographic changes, rising comorbidity rates, the difficulty of making accurate diagnoses, and poor access to treatment are some of the challenges faced by healthcare systems in the management of chronic pain. Among all the treatment options, opioids have played an increasingly important role in the control of moderate to severe pain (Goldberg and McGee, 2011; Majors-Foley, 2016; Andrews et al., 2018).

The main indication of strong opioids is for acute pain of moderate to severe intensity, especially for oncological or postoperative pain (World Health Organisation, 1996). Due to their known side effects, they must be monitored under the strict control of a healthcare professional (Labianca et al., 2012). Recent data have shown an increase in opioid consumption for the treatment of chronic non-cancer pain (CNCP), including osteoarthritis of the extremities and spondylarthritis as the most common pathologies (Agència de Qualitat i Avaluació Sanitàries de Catalunya, 2018). Although opioids are useful for alleviating pain, their use and side effects have not been thoroughly studied in the case of many pathologies that cause CNCP, and little evidence has been reported on the risk-benefit balance in these cases. Even so, many clinical practice guidelines maintain that these drugs should be second-line drugs (that is, administered under strict monitoring by a healthcare professional) due to their negative effects on cognition and their addictive potential (Kahan et al., 2011; Labianca et al., 2012; Manchikanti et al., 2012; Scottish Intercollegiate Guidelines Network (SIGN), 2013; Agència de Qualitat i Avaluació Sanitàries de Catalunya, 2018).

Given the generalised increase in consumption, various indicators of misuse (including addiction rates or overdose-related deaths) have been described in different contexts, and have revealed similar trends (Kalkman et al., 2019; Schieber et al., 2019). This trend has been observed on a large scale in the United States, the country with the most opioid users in the world: between 1999 and 2007, sales of opioid drugs increased by 149% (Manchikanti et al., 2013), and there were 399,230 overdose deaths (Scholl et al., 2018). As a result, in 2017 the US declared this issue a public health emergency and opioid abuse is still considered an epidemic today (US Department of Health and Human Services, n.d.).

Though less pronounced than in the United States, a similar trend has been observed in Europe in the last few years (Bosetti et al., 2019; European Monitoring Centre for Drugs and Drug Addiction, 2020). Increases in opioid use have also been recorded in Spain, where consumption rose by 88% between 2010 and 2018, with a notable increase in the use of strong opioids (Agencia Española de Medicamentos y Productos Sanitarios, n.d.). The same trend is observed in Catalonia, where total use of opioids between 2012 and 2016 increased by 45% (Pando et al., 2017). Among strong opioids, the rise in fentanyl use was particularly marked.

An issue that has not been widely addressed so far is the presence of inequalities in opioid use. The analysis of the social determinants of opioid use has been paid far less attention than clinical characteristics and age as the main determinants of its increase, despite the reports of ethnic, socioeconomic, gender, and geographical variations (Morden et al., 2014; Song, 2017; Santoro and Santoro, 2018; Friedman et al., 2019; Schieber et al., 2019; Park and Wu, 2020).

The aim of the present study is to analyse the geographical, socioeconomic, and gender determinants of the use of opioids for CNCP in Catalonia.

## Materials and Methods

### Design and Study Population

Multi-group ecological study using the health area (HA) as unit of analysis. In 2019, the Administrative Health Division divided Catalonia into 372 HAs. The entire Catalan population as of 2019 was considered as the study population, comprising a total of 7,653,845 inhabitants.

### Data Sources

Two data sources were used. The first was the Central Registry of Insured Persons, in order to obtain the reference population for 2019. This is an automated file that includes all residents in Catalonia and their sociodemographic characteristics (sex, age, HA of residence). The second was the Electronic Prescription Database, an administrative database providing data on outpatient dispensing, corresponding to the drugs dispensed by all pharmacy offices during the year 2019. It has been systematically validated by the healthcare system, which pays part of the prescription, and by the providers (i.e., the pharmacies) which carry out the billing. From this database, we obtained all the doses dispensed in defined daily doses (DDD) for each of the opioids selected according to age, sex, HA and medical diagnosis associated with the prescription. Drugs were all coded according to Anatomical Therapeutic Chemical (ATC) system (level 7) and medical diagnoses according to the Clinical Classification Software (CCS) level (HCUP CCS, 2017).

### Variables

#### Dependent Variable

The study included all opioid drugs authorised for analgesic purposes and available at community pharmacies in Spain in 2019. We distinguished between strong and weak opioids following the classification recorded at the 1961 Convention of Narcotic Drugs (Lande, 1962). Drugs were assigned to each category depending on their level of analgesia and dependency. Then, we listed the opioids as strong or weak according to their ATC7 codes: strong, morphine (N02AA01 and N02AA03), oxycodone (N02AA05 and N02AA55), phenylpiperidine derivatives (N02AB03 and N02AB02), tapentadol (N02AX06), and buprenorphine (N02AE01); and weak, codeine and combinations (N02AJ06, N02AJ07, and N02AJ08), and tramadol (N02AJ13, N02AJ14, N02AX02, and N02AX52).

The DDD/1,000 inhabitants per day is the main unit measure of the study. The DDD is a global standardised measure of drug consumption that corresponds to the assumed average maintenance dose per day for a drug used in its main indication among adults. DDDs are established and updated by the WHO (WHO Collaborating Centre for Drug Statistics, n. d.). The DDD/1,000 inhabitants per day measure provides a rough estimate of the proportion of the study population treated daily with a particular drug or group of drugs.

### Independent Variables

Socioeconomic variables: The composite socioeconomic index (ISC, Catalan acronym) was used to measure the socioeconomic level of each HA, and specifically the level of deprivation. The ISC includes mainly employment (unemployed rate and occupational status), social (educational level), economic (income level), health (hospitalization rate and preventable mortality) variables. It has shown a high correlation with healthcare use and morbidity in HA (Colls et al., 2020). Higher values on a range from 0 to 100 indicate higher levels of deprivation). We grouped the HAs into quartiles according to their ISC: Q1 (<34), Q2 (35–42), Q3 (43–50) and Q4 (>50). Of the 372 HAs in Catalonia, six were created after the last update of the ISC; in these cases, we assigned the ISC of the HA to which they previously belonged.

Sociodemographic variables: Sex, HA, and age of the opioid users, categorised into five groups: 0–14, 15–44, 45–64, 65–74, and 75 years or more.

Clinical variable: A clinical binary variable was constructed to reflect whether the opioid was prescribed for a cancer diagnosis: all cancer diagnoses included in the multi-level category CCS 2 (neoplasm), vs all other patients who did not have a cancer diagnosis (CNCP).

### Data Analysis

First, we performed a descriptive analysis of opioid users of Catalonia by age, socioeconomic status and sex.

Second, we determined the rate of opioid use (DDD/1,000 inhabitants per day) by drug type, sex, socioeconomic status, age group, and diagnosis. The use rate was calculated by using the dispensing data to obtain the total DDD according to the sociodemographic, socioeconomic and clinical variables and the reference population as a denominator. We then performed a descriptive analysis of the DDD/1,000 inhabitants per day by drug type.

Then, we conducted a direct standardization of DDD/1,000 inhabitants per day by age using the total population of Catalonia in 2019 as our reference population, for both sexes. To obtain a summary of all the information, the standardised values were displayed using maps of the HAs in Catalonia for both men and women.

Finally, robust Poisson models were run to test the association between opioid use (at DDD/1,000 inhabitants per day) and ISC quartiles by age and sex for both weak and strong opioid types. The models were adjusted for cancer diagnosis and age. Additional robust Poisson models were run, stratified by cancer diagnosis. From these models, we estimated the odds ratio (OR) with corresponding 95% confidence intervals (CI) and associated *p*-values.

The statistical analysis was carried out using the STATA 16.1 IC (StataCorp, n.d.) software, and the maps used to display the data were designed through the Instamaps app of the Cartographical and Geological Institute of Catalonia (Institut Cartografic Generalitat Catalunya, n.d.).

## Results

### Opioid Use in Catalonia

The total study population comprised 3,755,837 (49.07%) men and 3,898,009 (50.93%) women. Opioids were used by 218,354 males (36.44%) and 380,904 females (63.56%). (See Table 1 for the main characteristics of the study population).

**TABLE 1:** Descriptive characteristics of the population and opioid users of Catalonia, 2019.

	All population				Opioid users			
	Men		Women		Men		Women	
	**N**	(%)	**N**	(%)	**N**	(%)	**N**	(%)
Age (years)								
0–14	588,812	15.68	554,721	14.23	281	0.13	345	0.09
15–44	1,456,934	38.79	1,416,080	36.33	49,922	22.86	64,320	16.89
45–64	1,082,265	28.82	1,092,157	28.02	79,648	36.48	123,597	32.45
65–74	342,700	9.12	396,059	10.16	41,660	19.08	77,787	20.42
≥75	285,126	7.59	438,991	11.26	46,843	21.45	114,855	30.15
ISC^a^ (quartile)								
Q1 (least deprived)	1,082,845	28.83	1,185,533	30.41	47,969	21.97	91,876	24.12
Q2	927,086	24.68	951,677	24.41	54,828	25.11	93,623	24.58
Q3	840,638	22.38	851,176	21.84	53,147	24.34	89,224	23.42
Q4 (most deprived)	905,268	24.10	909,622	23.34	62,410	28.58	106,181	27.88
Total	3,755,837	49.07	3,898,009	50.93	218,354	36.44	380,904	63.56

a ISC: Composite socioeconomic index.

Table 2 presents the DDD/1,000 inhabitants per day of strong and weak opioids according to age, ISC, and diagnosis. Median use of all opioids was 11.66 (Interquartile Range (IQR) 3.61–21.78) DDD/1,000 inhabitants per day; for strong opioids, it was 4.72 (IQR 1.47–12.4) DDD/1,000 inhabitants per day, and for weak opioids, it was 7.03 (IQR 2.14–15.41). Use of both opioid types was higher in women than in men. For both types of drugs and for both sexes, use rates were higher in HAs with lower ISC.

**TABLE 2:** Opioid use (DDD/1,000 inhabitants per day) according to composite socioeconomic index, age, sex, and clinical diagnosis according to type of drug in Catalonia, 2019.

	DDD/1,000 inhabitants per day			
	Weak opioids		Strong opioids	
	Men	Women	Men	Women
Age (years old)				
0–14	0.03	0.04	0.01	0.00
15–44	1.77	2.31	0,61	0.67
45–64	5.39	10.01	3.24	4.66
65–74	10.45	21.68	5.88	11.67
≥75	15.40	31.22	11.06	32.37
ISC^a^ (quartile)				
Q1 (least deprived)	3.24	7.02	2.02	5.21
Q2	4.35	9.32	2.60	6.48
Q3	4.90	10.40	2.77	6.89
Q4 (most deprived)	5.23	11,46	2.86	7.24
Cancer diagnosis				
Yes	0.11	0.13	0.65	0.60
No	4.26	9.24	1.90	5.78

a ISC: Composite socioeconomic index.

Opioid use increased with age, more so among women than among men. For instance, the DDD/1,000 inhabitants per day of strong opioids in women over 75 years old was 177% higher than in women between 65 and 74, compared with a corresponding increase in men across the same age of approximately 88%.

Patients who used opioids to treat CNCP showed higher use rates, for both weak and strong types. Again, there was a clear difference of DDD/1,000 inhabitants per day according to sex, with higher opioid use in women (see Table 2).

**FIGURE 1:**
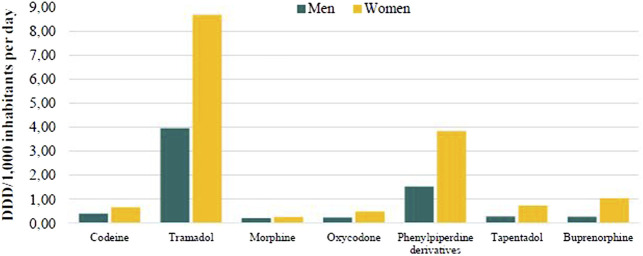
Opioid use (DDD/1,000 inhabitants per day) by sex and type of drug in Catalonia, 2019.

Figure 1 also shows higher use in women than in men, for all kinds of opioids. In both sexes, tramadol was the most used and morphine the least.

### Inequalities in Opioid Use

**FIGURE 2:**
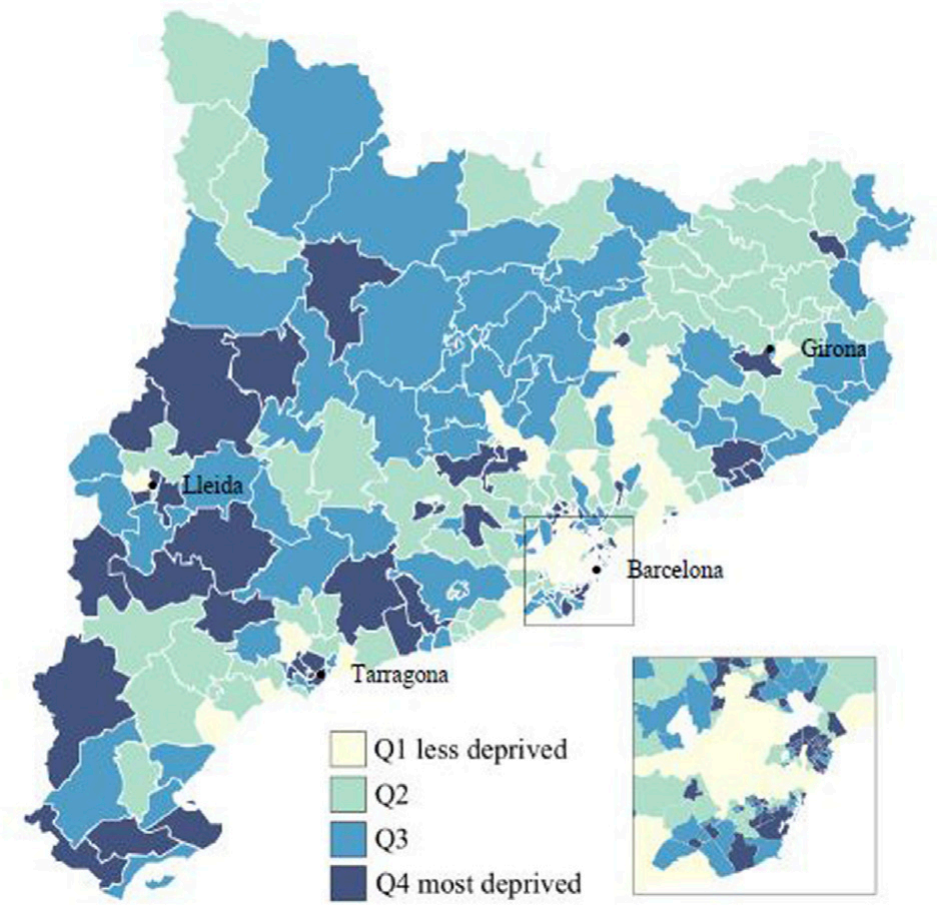
Composite socioeconomic index (ISC) in quartiles for the health areas in Catalonia, 2019.

Figure 2 shows the distribution of HAs according to the ISC quartiles. The most advantaged HAs were located in the city of Barcelona, in the other three main urban regions (Girona, Tarragona, and Lleida), and in Barcelona’s northern metropolitan area. Nonetheless, it was in the metropolitan area where the greatest inequalities were observed.

**FIGURE 3:**
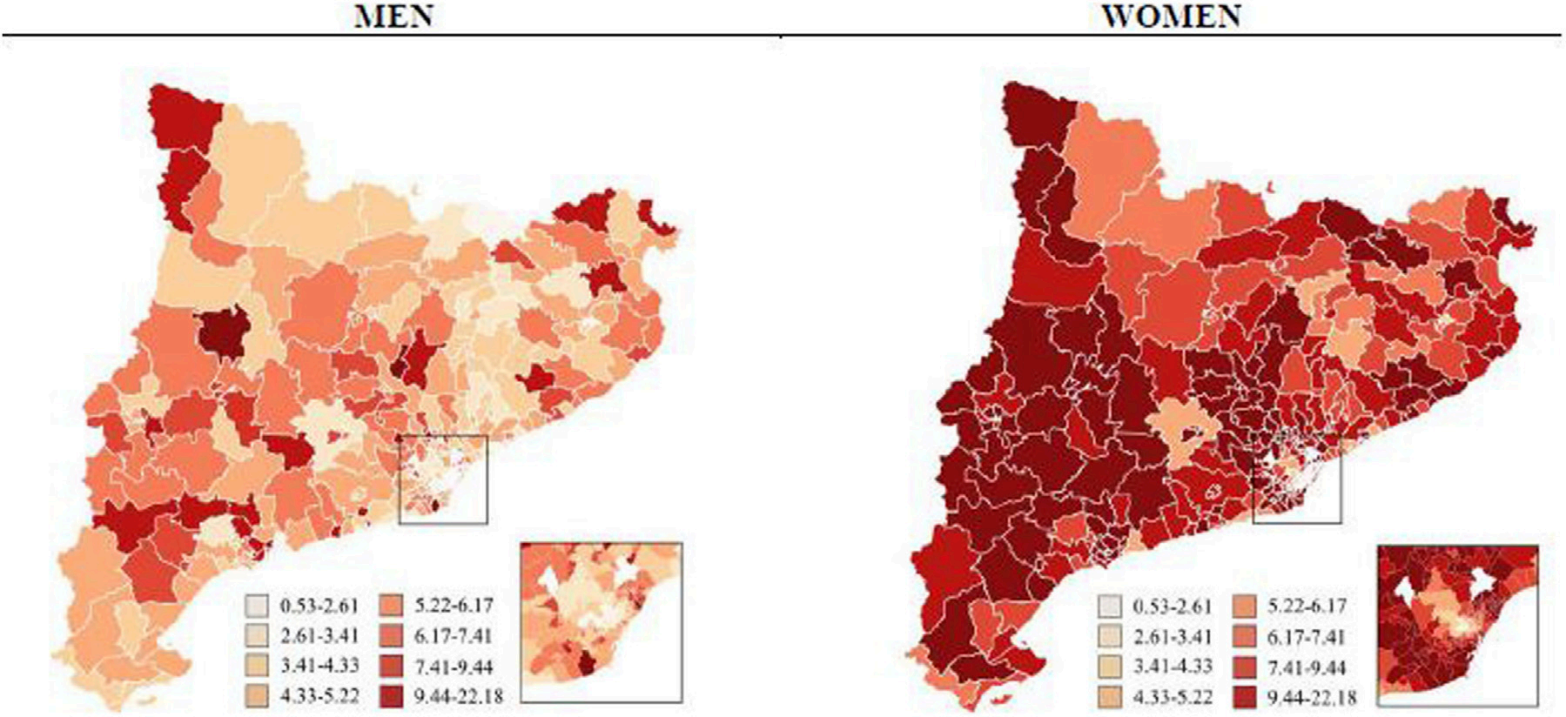
Weak opioids use (DDD/1,000 inhabitants per day standardized by age) by sex and health area in Catalonia, 2019.

**FIGURE 4:**
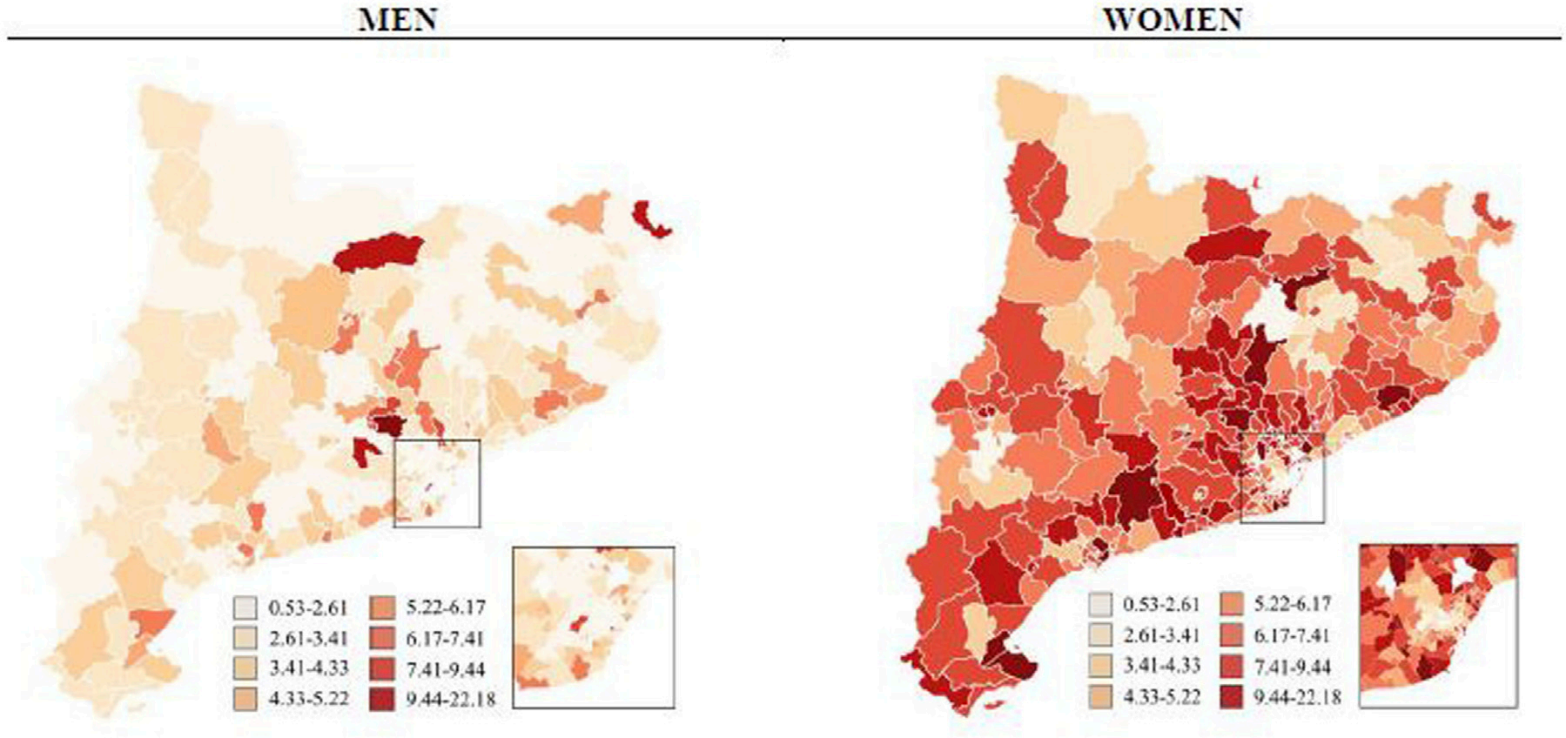
Strong opioids use (DDD/1,000 inhabitants per day standardized by age) by sex and health area in Catalonia, 2019.

Figures 3, 4 display comparative maps of the distribution of opioid use in DDD/1,000 inhabitants per day standardised by age for each HA, based on sex and drug type. The maps show a visual correlation: the lower the ISC, the lower the use of opioids. This was particularly clear in the HAs of the city of Barcelona. In women, the use of weak opioids tended to be higher in the HAs of Lleida and Tarragona, while that of strong opioids was higher in the Catalan coastal area. In men, the distribution was less homogeneous and no clear visual pattern of use was observed.

The largest gap in the DDD/1,000 inhabitants per day in the different HAs was observed in the use of weak opioids in women (Figure 3), where the HA with the lowest rate was 2.34 DDD/1,000 inhabitants per day compared to 22.18 in the HA with the highest rate.

### Factors Associated With Opioid Use

Tables 3, 4 show the OR obtained from the robust Poisson models. There was a statistically significant negative association between use rates in the most deprived HAs and those of the most advantaged ones, in so far as the use in the most deprived HAs was higher. This was seen in all ages, especially in the age 45–64 bracket where weak opioid use in women was 124% higher in the most deprived HAs than in the most affluent ones (Q4, OR = 2.24, 95%CI = 2.12, 2.37, *p* < 0.001).

**TABLE 3:** Odds ratio (OR) of weak opioids use by sex, age and composite socioeconomic index (quartiles) adjusted for cancer diagnosis and age in Catalonia, 2019.

	Men			Women		
	OR	95% CI	p-value	OR	95% CI	p-value
0–14 years old^a^	nc	nc	nc	nc	nc	nc
15–44 years old						
Q1 (least deprived)	1.00	—	—	1.00	—	—
Q2	1.56	(1.34–1.82)	<0.001^b^	1.64	(1.45–1.86)	<0.001^b^
Q3	1.70	(1.46–1.98)	<0.001^b^	1.82	(1.61–2.06)	<0.001^b^
Q4 (most deprived)	2.03	(1.76–2.34)	<0.001^b^	2.11	(1.87–2.38)	<0.001^b^
45–64 years old						
Q1 (least deprived)	1.00	—	—	1.00	—	—
Q2	1.51	(1.40–1.64)	<0.001^b^	1.60	(1.51–1.70)	<0.001^b^
Q3	1.77	(1.64–1.92)	<0.001^b^	1.90	(1.79–2.01)	<0.001^b^
Q4 (most deprived)	2.08	(1.93–2.25)	<0.001^b^	2.24	(2.12–2.37)	<0.001^b^
65–74 years old						
Q1 (least deprived)	1.00	—	—	1.00	—	—
Q2	1.40	(1.27–1.55)	<0.001^b^	1.48	(1.39–1.58)	<0.001^b^
Q3	1.58	(1.43–1.74)	<0.001^b^	1.66	(1.56–1.77)	<0.001^b^
Q4 (most deprived)	1.78	(1.62–1.95)	<0.001^b^	1.91	(1.80–2.03)	<0.001^b^
≥75 years old						
Q1 (least deprived)	1.00	—	—	1.00	—	—
Q2	1.21	(1.11–1.32)	<0.001^b^	1.20	(1.15–1.26)	<0.001^b^
Q3	1.35	(1.24–1.47)	<0.001^b^	1.28	(1.22–1.34)	<0.001^b^
Q4 (most deprived)	1.41	(1.30–1.54)	<0.001^b^	1.38	(1.31–1.44)	<0.001^b^

a The results for the 0–14 age group are not conclusive, due to the low use in this group.

b Statistical significance at ≤0.05%.

**TABLE 4:** Odds ratio (OR) of strong opioids use by sex, age, and composite socioeconomic index (quartiles), adjusted for cancer diagnosis and age in Catalonia, 2019.

	Men			Women		
	OR	95% CI	p-value	OR	95% CI	p-value
0–14 years old^a^	nc	nc	nc	nc	nc	nc
15–44 years old						
Q1 (least deprived)	1.00	(1.00–1.00)	—	1.00	(1.00–1.00)	—
Q2	2.57	(2.03–3.26)	<0.001^b^	1.18	(0.96–1.44)	0.1079
Q3	2.50	(1.96–3.18)	<0.001^b^	1.51	(1.24–1.83)	<0.001^b^
Q4 (most deprived)	2.37	(1.86–3.01)	<0.001^b^	1.51	(1.25–1.82)	<0.001^b^
45–64 years old						
Q1 (least deprived)	1.00	(1.00–1.00)	—	1.00	(1.00–1.00)	—
Q2	1.30	(1.18–1.43)	<0.001^b^	1.44	(1.32–1.56)	<0.001^b^
Q3	1.47	(1.33–1.62)	<0.001^b^	1.74	(1.61–1.89)	<0.001^b^
Q4 (most deprived)	1.60	(1.45–1.75)	<0.001^b^	2.06	(1.90–2.23)	<0.001^b^
65–74 years old						
Q1 (least deprived)	1.00	(1.00–1.00)	—	1.00	(1.00–1.00)	—
Q2	1.41	(1.24–1.60)	<0.001^b^	1.56	(1.44–1.70)	<0.001^b^
Q3	1.65	(1.45–1.87)	<0.001^b^	1.68	(1.54–1.82)	<0.001^b^
Q4 (most deprived)	1.56	(1.37–1.77)	<0.001^b^	1.86	(1.71–2.02)	<0.001^b^
≥75 years old						
Q1 (least deprived)	1.00	(1.00–1.00)	—	1.00	(1.00–1.00)	—
Q2	1.19	(1.08–1.32)	<0.001^b^	1.25	(1.19–1.31)	<0.001^b^
Q3	1.28	(1.15–1.41)	<0.001^b^	1.27	(1.21–1.33)	<0.001^b^
Q4 (most deprived)	1.34	(1.21–1.48)	<0.001^b^	1.33	(1.26–1.39)	<0.001^b^

a The results for the 0–14 age group are not conclusive, due to the low use in this group.

b Statistical significance at ≤0.05%.

Furthermore, a steeper gradient was observed in women than in men, for all ages. This gap was particularly noticeable in the 45–64 years age range, for both strong and weak opioids. For example, use of strong opioids was 106% higher in women in Q4 areas than in their peers in Q1 areas (Q4, OR = 2.06, 95%CI = 1.90, 2.23, *p* < 0.001), while in men the difference was 60% (Q4, OR = 1.60, 95%CI = 1.45, 1.75, *p* < 0.001). However, the socioeconomic gradients observed between areas were, in most cases, steeper for weak opioids than strong opioids, for all age ranges and for both sexes.

Tables 5, 6 present the results of the OR obtained from the models stratified by cancer diagnosis. The results were similar to those observed in the previous model. Although not all coefficients reached statistical significance, in most cases, the associations were significant for the most disadvantaged HAs. However, it should be noted that generally the results for cancer-related use were not statistically significant, whereas results for opioids prescribed for other causes did reach significance.

**TABLE 5:** Odds ratio (OR) of weak opioids use by sex, age, composite socioeconomic index (quartiles), and cancer diagnosis adjusted for age in Catalonia, 2019.

	Cancer						No cancer					
	Men			Women			Men			Women		
	OR	95% CI	p-value	OR	95% CI	p-value	OR	95% CI	p-value	OR	95% CI	p-value
0–14 years old^a^	nc	nc	nc	Nc	nc	nc	nc	nc	nc	nc	nc	nc
15–44 years old												
Q1 (least deprived)	1.00	—	—	1.00	—	—	1.00	—	—	1.00	—	—
Q2	1.48	(0.27–8.02)	0.649	0.98	(0.36–2.65)	0.961	1.56	(1.34–1.82)	<0.001^b^	1.65	(1.46–1.87)	<0.001^b^
Q3	1.15	(0.18–7.19)	0.881	1.30	(0.50–3.38)	0.587	1.70	(1.46–1.98)	<0.001^b^	1.83	(1.61–2.07)	<0.001^b^
Q4 (most deprived)	1.88	(0.38–9.37)	0.440	1.36	(0.54–3.44)	0.510	2.03	(1.76–2.35)	<0.001^b^	2.12	(1.88–2.40)	<0.001^b^
45–64 years old												
Q1 (least deprived)	1.00	—	—	1.00	—	—	1.00	—	—	1.00	—	—
Q2	1.31	(0.77–2.22)	0.324	1.31	(0.83–2.05)	0.249	1.52	(1.40–1.65)	<0.001^b^	1.61	(1.52–1.70)	<0.001^b^
Q3	1.35	(0.79–2.33)	0.275	1.61	(1.04–2.52)	0.034^b^	1.78	(1.64–1.93)	<0.001^b^	1.90	(1.80–2.01)	<0.001^b^
Q4 (most deprived)	2.02	(1.23–3.32)	0.005^b^	1.81	(1.18–2.79)	0.007^b^	2.08	(1.93–2.25)	<0.001^b^	2.25	(2.13–2.38)	<0.001^b^
65–74 years old												
Q1 (least deprived)	1.00	—	—	1.00	—	—	1.00	—	—	1.00	—	—
Q2	1.15	(0.70–1.90)	0.583	1.34	(0.79–2.28)	0.274	1.41	(1.28–1.56)	<0.001^b^	1.49	(1.40–1.58)	<0.001^b^
Q3	1.01	(0.60–1.73)	0.959	1.57	(0.93–2.64)	0.090	1.60	(1.45–1.76)	<0.001^b^	1.66	(1.56–1.77)	<0.001^b^
Q4 (most deprived)	1.49	(0.92–2.41)	0.109	1.57	(0.93–2.63)	0.089	1.79	(1.63–1.97)	<0.001^b^	1.92	(1.80–2.03)	<0.001^b^
≥75 years old												
Q1 (least deprived)	1.00	—	—	1.00	—	—	1.00	—	—	1.00	—	—
Q2	1.24	(0.79–1.96)	0.356	1.16	(0.78–1.73)	0.454	1.21	(1.11–1.32)	<0.001^b^	1.20	(1.15–1.26)	<0.001^b^
Q3	1.36	(0.86–2.15)	0.194	1.05	(0.69–1.60)	0.826	1.35	(1.23–1.47)	<0.001^b^	1.28	(1.22–1.35)	<0.001^b^
Q4 (most deprived)	1.32	(0.83–2.08)	0.238	1.17	(0.78–1.75)	0.461	1.42	(1.30–1.55)	<0.001^b^	1.38	(1.32–1.45)	<0.001^b^

a The results for the 0–14 age group are not conclusive, due to the low use in this group.

b Statistical significance at ≤0.05%.

**TABLE 6:** Odds ratio (OR) of strong opioids use by sex, age, composite socioeconomic index (quartiles), and cancer diagnosis adjusted for age in Catalonia, 2019.

	Cancer						No cancer					
	Men			Women			Men			Women		
	OR	95% CI	p-value	OR	95% CI	p-value	OR	95% CI	p-value	OR	95% CI	p-value
0–14 years old^a^	nc	nc	Nc	nc	nc	nc	nc	nc	nc	nc	nc	nc
15–44 years old												
Q1 (least deprived)	1.00	—	—	1.00	—	—	1.00	—	—	1.00	—	—
Q2	9.41	(4.63–19.12)	<0.001^b^	1.15	(0.71–1.86)	0.5820	1.92	(1.48–2.50)	<0.001^b^	1.18	(0.95–1.47)	0.1298
Q3	8.33	(4.06–17.09)	<0.001^b^	1.33	(0.82–2.15)	0.2432	1.94	(1.49–2.53)	<0.001^b^	1.54	(1.25–1.90)	<0.001^b^
Q4 (most deprived)	3.43	(1.59–7.43)	0.0018^b^	1.76	(1.13–2.73)	0.0126^b^	2.27	(1.76–2.92)	<0.001^b^	1.46	(1.18–1.80)	0.0004^b^
45–64 years old												
Q1 (least deprived)	1.00	—	—	1.00	—	—	1.00	—	—	1.00	—	—
Q2	1.05	(0.88–1.26)	0.5633	1.01	(0.84–1.22)	0.8849	1.42	(1.26–1.60)	<0.001^b^	1.57	(1.43–1.72)	<0.001^b^
Q3	1.07	(0.89–1.28)	0.4853	1.02	(0.84–1.24)	0.8102	1.66	(1.48–1.87)	<0.001^b^	1.96	(1.79–2.15)	<0.001^b^
Q4 (most deprived)	1,29	(1.08–1.53)	0.0043^b^	1.46	(1.22–1.74)	<0.001^a^	1.75	(1.56–1.96)	<0.001^b^	2.24	(2.05–2.45)	<0.001^b^
65–74 years old												
Q1 (least deprived)	1.00	—	—	1.00	—	—	1.00	—	—	1.00	—	—
Q2	1.17	(0.94–1.46)	0,1601	1.17	(0.92–1.49)	0.1958	1.54	(1.32–1.80)	<0.001^b^	1.63	(1.49–1.78)	<0.001^b^
Q3	1.65	(1.34–2.03)	<0.001^b^	1.44	(1.14–1.83)	0.0021^b^	1.65	(1.41–1.93)	<0.001^b^	1.71	(1.57–1.88)	<0.001^b^
Q4 (most deprived)	1.40	(1.13–1.74)	0.0021^b^	1.43	(1.13–1.81)	0.0025^b^	1.65	(1.41–1.93)	<0.001^b^	1.93	(1.76–2.11)	<0.001^b^
≥75 years old												
Q1 (least deprived)	1.00	—	—	1.00	—	—	1.00	—	—	1.00	—	—
Q2	1.03	(0.82–1.29)	0.8156	0.94	(0.78–1.14)	0.5306	1.23	(1.10–1.38)	0.0003^b^	1.27	(1.21–1.33)	<0.001^b^
Q3	1.25	(1.00–1.56)	0.0545	0.93	(0.76–1.14)	0.4753	1.28	(1.14–1.44)	<0.001^b^	1.29	(1.23–1.36)	<0.001^b^
Q4 (most deprived)	1.38	(1.11–1.72)	0.0033^b^	1.01	(0.83–1.22)	0.9347	1.32	(1.18–1.48)	<0.001^b^	1.35	(1.28–1.41)	<0.001^b^

a The results for the 0–14 age group are not conclusive, due to the low use in this group.

b Statistical significance at ≤0.05%.

As with the main model, a clear socioeconomic gradient across areas was observed for the use of opioids to treat CNCP. This gradient was less steep in prescriptions for cancer pain control.

## Discussion

We assessed the results of this study from two perspectives: first, focusing on the socioeconomic dimension, and second, focusing on gender.

This study reveals a strong geographical and socioeconomic gradient in opioid use in Catalonia. The results showed that the DDD/1,000 inhabitants per day was 10 times higher in the HA with the highest use than in the one with the lowest. The most striking case was the use of weak opioids in women, where the lowest rate (2.34 DDD/1,000 inhabitants per day) was recorded in the Barcelona 5-C HA (an affluent district of the city), and the highest rate (22.18) was recorded in the Badalona 5 HA (a deprived area). In addition, the descriptive maps show that in metropolitan areas the inequalities were more evident, both in terms of socioeconomic status and in opioid use. This finding was especially notable in the metropolitan area of Barcelona, but it was also observed in the cities of Girona, Lleida, and Tarragona, three of the region’s major urban areas.

These socioeconomic differences in opioid use could be explained by two factors. The first possible explanation concerns the differences between the individual socioeconomic characteristics of the inhabitants of the different HAs. Previous literature reports have highlighted the wide-ranging impact of socioeconomic conditions on the population’s health (García-Altés et al., 2018; Kivimäki et al., 2020). In the specific case of CNCP, the risk of suffering this type of pain is higher in unfavourable socioeconomic conditions (Riskowski, 2014; Grol-Prokopczyk, 2017; Jonsdottir et al., 2019), a circumstance that may increase opioid prescription. The second explanation relates to the differences in the characteristics of each HA. Factors such as the concentration of poverty and social segregation, the physical structure of the environment (e.g., adaptability for people with disabilities, accessibility to public transport, etc.) or the network of public facilities may have a decisive influence on the prevalence of morbidities and pain (Marí-Dell’Olmo et al., 2016; Rocha et al., 2017).

Other important issues for debate are environmental or individual differences such as the access to supplementary treatments (such as additional physiotherapy or exercise programmes not covered by public health services) and the access to pain treatment specialists in different HAs. These treatments may be less accessible to some people, either for economic reasons, lack of time, or because they cannot prioritize their own care. Differences between HAs may also be driven by the availability of multidisciplinary services in pain management. In this context, the results of the study suggest that opioid analgesics have become a fast and economical way for the Catalan health system to treat CNCP. In addition, opioid management differs according to the approaches of the medical teams and the guidelines in place in each HA, which may explain some of the differences in use patterns. Previous studies in the United Kingdom, which like Catalonia has a universal healthcare system, have identified similar risk factors in opioid prescribing, including the lack of time spent on dedicated patient care and socioeconomic deprivation (Curtis et al., 2019). In contrast, one study in Mexico showed that areas with a higher socio-economic status had higher rates of opioid dispensing after adjusting for palliative care need. Factors such as the coexistence of different health systems and differences in access both to medicines and to health services may be the main reasons for these disparities, with the most vulnerable areas being the ones with the lowest provision of these services (Goodman-Meza et al., 2021). The studies just mentioned and the present one all highlight the importance of the health system as a guarantor of equitable access to pain treatment and the use of opioid drugs. Another noteworthy finding is that the socioeconomic gradient is more pronounced in intermediate ages, especially in the 45–64 years range, where in many cases the increase in use between the most and the least favoured areas exceeds 100%.

This study found a more pronounced socioeconomic gradient in the prescription of weak than of strong opioids. This may be due to the fact that the prescription of strong opioids tends to be more protocolised and controlled, which may reduce the differences in consumption between socioeconomic groups. Likewise, in the prescription of opioids for a cancer diagnosis, socioeconomic status has a much lower impact than in the case of a diagnosis of non-oncological pain. There are two possible explanations for this. First, cancer is a particularly sensitive issue for the healthcare system and its treatment follows clearly established guidelines, with the result that the influence of socioeconomic factors is probably less important than in other situations. Second, the data on drug prescription used in this study corresponded to drugs dispensed at a community pharmacy office; it was not possible to obtain data on consumption in the hospital or long-term care setting. Despite the fact that socioeconomic status has a lesser impact on the prescription of opioids for oncological pain, our results indicated some notably high values in the most disadvantaged HAs, especially in the prescription of strong opioids. On this point, previous research has pointed out that an individual’s socioeconomic status affects their risk of developing cancer (Mihor et al., 2020).

From a gender perspective, the results show huge differences in the consumption of opioids between men and women. The issue of gender inequalities in health has been extensively studied; women have been found to present worse indicators of perceived health and higher prevalence of certain pathologies (Mauvais-Jarvis et al., 2020) and pain (Fillingim et al., 2009; Andrews et al., 2018; Sylwander et al., 2020). Moreover, women tend to make greater use of health services (García-Altés et al., 2018; Mihor et al., 2020). Regarding opioid consumption, areas with lower Gender Development Index scores have the worst results in analgesic management indicators. Women are prescribed more opioids than men but make fewer visits to specialist doctors (Chilet-Rosell et al., 2013). Differences in attitudes to opioid management between healthcare units and professionals have also been reported (Green et al., 2003; Perelló Bratescu et al., 2020). What remains unclear is the relative contribution of biological sex to pain mechanisms and its distribution and the contribution of gender (Fillingim et al., 2009). A gender perspective approach is needed to accumulate more evidence and clarify its social influence to chronic pain and its management (Serdarevic et al., 2017; Mauvais-Jarvis et al., 2020).

In interpreting the results, the greater proportion of women in the population should be borne in mind. This difference becomes wider at more advanced ages, as women in the over-75 age group outnumber men by 50%. However, data on individual opioid users showed that, in the same population, there were more female than male users in absolute and relative terms for both strong and weak opioids, thus maintaining the gender differences.

The results of the study show that the socioeconomic gradient in opioid use is much more pronounced in women than in men. Gender should be established as a key social factor in opioid consumption for two reasons: the gender bias in the health system and the poorer health status of women, partly due to a higher manifestation of pain symptoms and partly due to their less favourable socioeconomic status. These dimensions are intersectional and explain, among other things, the large differences observed between men and women (Serdarevic et al., 2017; Mauvais-Jarvis et al., 2020).

This study has some limitations. First of all, as it is an ecological study, it is impossible to determine the effect that the various factors have on the use of opioid analgesics at the individual level. Also, within an HA with an assigned ISC, there might be individuals with very different characteristics. Second, the 1 year time period used in the study does not allow us to determine trends in opioid use. Third, cancer prevalence and the patients-per-physician ratio in each HA were not taken into account. Finally, the data corresponds to the drugs dispensed by the public health system in pharmacies. Therefore, we do not have data on private consumption (i.e., through private insurance or other sources), or data on hospitals or long-term care prescriptions.

The data on opioid prescribing should also be interpreted with care. A high opioid use does not necessarily reflect inadequate pain control management; it may represent a satisfactory response of the health system to the requirements of the population. The aim of this study is to highlight the gap existing between use rates due to different socioeconomic and sociodemographic factors, and is certainly not to criticise legitimate medical uses.

Despite the limitations, this study presents a number of strengths. We used administrative medical databases that include records of all public healthcare services and prescriptions dispensed for the whole Catalan population. These sources provided valuable information and are increasingly used as a robust tool in public health research.

To conclude, our results shed light on the social differences existing between geographical areas in the management of opioid use in CNCP. Our findings calls for stronger action to promote best practices in prescribing for chronic pain and to reduce socioeconomic and gender variation between geographical areas. This study also provides a model for routine monitoring of opioid prescribing in order to promote interventions able to reduce high-dose consumption in identified HAs.

## Data Availability

The original contributions presented in the study are available through the Instamaps website. https://www.instamaps.cat/visor.html?businessid=586a401e5496f46278c33dbd8360a150&3D=false#, further inquiries can be directed to the corresponding author.
